# Behavioral destabilization induced by the selective serotonin reuptake inhibitor fluoxetine

**DOI:** 10.1186/1756-6606-4-12

**Published:** 2011-03-16

**Authors:** Katsunori Kobayashi, Yumiko Ikeda, Hidenori Suzuki

**Affiliations:** 1 Department of Pharmacology, Nippon Medical School, 1-1-5 Sendagi, Bunkyo-ku, Tokyo 113-8602, Japan; 2 Japan Science and Technology Agency, Core Research for Evolutional Science and Technology, Saitama 332-0012, Japan

## Abstract

**Background:**

Selective serotonin reuptake inhibitors (SSRIs) are widely used to treat mood and anxiety disorders. However, neuronal bases for both beneficial and adverse effects of SSRIs remain poorly understood. We have recently shown that the SSRI fluoxetine can reverse the state of maturation of hippocampal granule cells in adult mice. The granule cell "dematuration" is induced in a large population of granule cells, and greatly changes functional and physiological properties of these cells. Here we show that this unique form of neuronal plasticity is correlated with a distinct change in behavior of mice.

**Results:**

We chronically treated adult male mice with fluoxetine, and examined its effect on several forms of behavior of mice. During fluoxetine treatments, mice showed a marked increase in day-to-day fluctuations of home cage activity levels that was characterized by occasional switching between hypoactivity and hyperactivity within a few days. This destabilized cage activity was accompanied by increased anxiety-related behaviors and could be observed up to 4 weeks after withdrawal from fluoxetine. As reported previously, the granule cell dematuration by fluoxetine includes a reduction of synaptic facilitation at the granule cell output, mossy fiber, synapse to the juvenile level. Mossy fiber synaptic facilitation examined electrophysiologically in acute hippocampal slices also remained suppressed after fluoxetine withdrawal and significantly correlated with the fluctuation of cage activity levels in individual mice. Furthermore, in mice lacking the 5-HT_4 _receptor, in which the granule cell dematuration has been shown to be attenuated, fluoxetine had no significant effect on the fluctuation of cage activity levels.

**Conclusions:**

Our results demonstrate that the SSRI fluoxetine can induce marked day-to-day changes in activity levels of mice in the familiar environment, and that the dematuration of the hippocampal granule cells is closely associated with the expression of this destabilized behavior. Based on these results, we propose that the granule cell dematuration can be a potential cellular basis underlying switching-like changes in the behavioral state associated with SSRI treatments.

## Background

Selective serotonin reuptake inhibitors (SSRIs) have been commonly used to treat mood and anxiety disorders, while some severe adverse effects have been reported [1,2]. Although SSRIs can immediately change extracellular levels of serotonin in the central nervous system, therapeutic effects of these drugs usually require weeks of treatments [3]. Some of adverse psychiatric effects of SSRIs also emerge with a delayed onset during chronic treatments or even after withdrawal of the drugs [4,5]. Thus, adaptive or plastic changes in the central nervous system are likely to be involved in adverse effects as well as therapeutic effects of SSRIs. In experimental animals, SSRIs and other antidepressant drugs can generally facilitate adult neurogenesis in the dentate gyrus of the hippocampus, and this process has been suggested to underlie some of behavioral effects of these drugs [6-8]. The facilitatory effect on the adult neurogenesis usually requires a few weeks of administration, which could explain the delayed manifestation of the effects of the drugs at the behavioral level. However, it remains unknown how the facilitated neurogenesis leads to modifications of hippocampal functioning that can cause robust changes in behavior. We have recently shown that chronic treatments with fluoxetine, a widely used SSRI, can reverse the established state of maturation of the dentate granule cell in adult mice [9]. The change in the state of the granule cell maturation gradually develops over the course of the fluoxetine treatment for a few weeks and is manifested as marked changes in physiological and functional properties of the granule cell that include neuronal excitability, activity-dependent synaptic modifications, and immediate early gene expression [9]. Since this novel form of neuronal plasticity is induced in a large population of the dentate granule cells, it is supposed to have a substantial impact on the operation of hippocampal neuronal circuits and probably on hippocampus-dependent regulation of behaviors. In the present study, we analyzed changes in behaviors of mice treated with fluoxetine in a regimen that is sufficient for the induction of granule cell dematuration, and examined the association between observed behavioral changes and the granule cell dematuration.

## Results

### Destabilization of home cage activity by chronic fluoxetine

**Figure 1:**
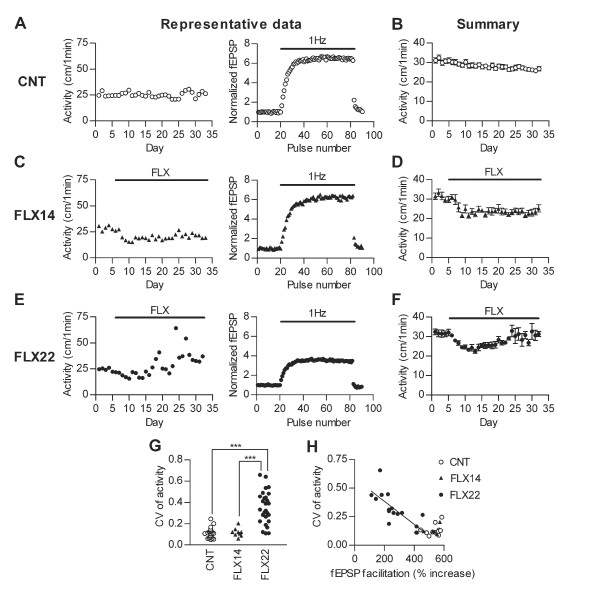
**Fluoxetine destabilizes home cage activity**. (**A**) Representative data showing nocturnal locomotor activity levels in home cage (left) and frequency facilitation at the mossy fiber synapse induced by 1 Hz stimulation (right) in a control (CNT) mouse. (**B**) Averaged data of nocturnal activity levels in control mice. (**C**) Representative nocturnal activity and synaptic facilitation, and (**D**) averaged nocturnal activity in mice treated with 14 mg/kg/day fluoxetine (FLX14). (**E**) Representative nocturnal activity and synaptic facilitation, and (**F**) averaged nocturnal activity in mice treated with 22 mg/kg/day fluoxetine (FLX22). Representative behavioral and electrophysiological data were obtained from the same mice. Averaged data are presented as mean ± s.e.m. (**G**) Coefficient of variation (CV) of activity levels during last 2 weeks of treatments. FLX22 (*n *= 29) mice showed larger variation than control (*n *= 27, rank sum difference = 29.48, *p *< 0.0001) and FLX14 (*n *= 9, rank sum difference = 27.11, *p *< 0.0001). There was no significant difference between control and FLX14 (rank sum difference = 2.37, *p *> 0.05). (**H**) CV of activity was negatively correlated with the magnitude of frequency facilitation at the mossy fiber synapse in FLX22 mice (*n *= 16, *p *= 0.0007, r^2 ^= 0.569). Each symbol represents a single mouse.

**Figure 2:**
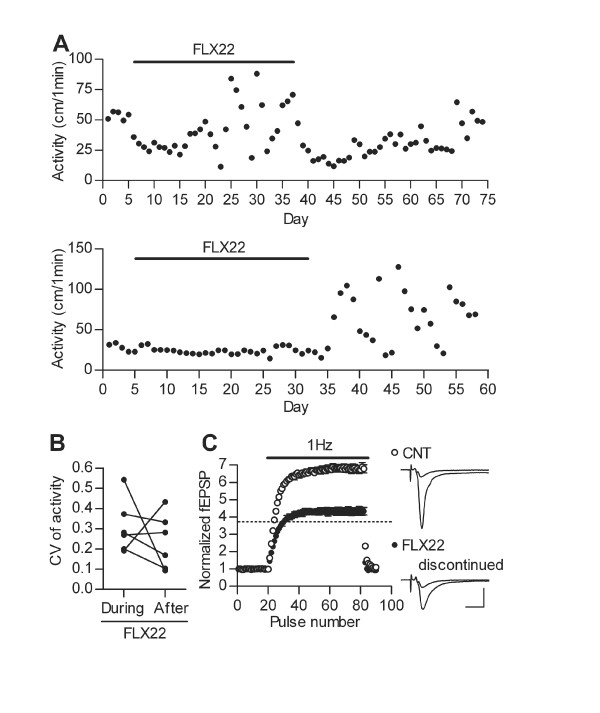
**Home cage activity and synaptic transmission after discontinuation of fluoxetine administration**. (**A**) Sample data showing effects of FLX22 administration and withdrawal on nocturnal activity in two different mice. (**B**) CV of activity during FLX22 treatments and after discontinuation of the treatments. Data points connected by lines were from the same mice. (**C**) Pooled data showing reduced 1 Hz frequency facilitation at the mossy fiber synapse 4 weeks after discontinuation of fluoxetine treatments (*n *= 6 each, U = 0, *p *= 0.0022). The dotted line shows the average level of facilitation in mice treated with FLX22 for 4 to 5 weeks without discontinuation. Sample recordings are averages of 15 consecutive fEPSPs before and during 1 Hz stimulation. Scale bar: 10 ms, 0.5 mV. Data are presented as mean ± s.e.m.

Fluoxetine was applied for 4 weeks at a dose of 14 or 22 mg/kg/day. Our previous study showed that fluoxetine induces dematuration of the dentate granule cells at 22 mg/kg/day, but not at 14 mg/kg/day [9]. In order to assess a behavioral correlate of the granule cell dematuration, we analyzed effects of both doses of fluoxetine on several forms of behaviors of mice. First, activity levels of mice in their home cages were continuously monitored during the treatment (Figure 1A-1F). In some of these mice, electrophysiological recordings were also performed after monitoring cage activity to assess the state of the granule cell maturation (Figure 1A, 1C and 1E). In control mice, the home cage activity levels were quite stable throughout the course of experiments (Figure 1A left) except for a gradual small decline as seen in the averaged data (Figure 1B). After starting administration of fluoxetine at 14 mg/kg/day (FLX14), the activity level of mice was initially decreased, but remained stable thereafter (Figure 1C left and 1D). In contrast, fluoxetine at 22 mg/kg/day (FLX22) caused a distinct change in the home cage activity. Mice treated with FLX22 also exhibited an initial decrease in activity levels (Figure 1E left and 1F). In about 2 weeks of treatments, however, they started showing a marked day-to-day fluctuation of activity levels (Figure 1E left) that was accompanied by occasional switching from hypoactivity to hyperactivity and vice versa in a few days (see also Figure 2A). To quantify this fluctuation of home cage activity, we calculated the coefficient of variation (CV) of activity levels during the last 2 weeks of the treatments. There was a highly significant difference in CV between FLX22 and other groups (Figure 1G). The mean activity level during the same period tended to be higher in FLX22-treated mice (see Figure 1B, 1D and 1F), but there was no statistically significant difference between groups (*p *> 0.05, rank sum difference = 4.327 for control vs. FLX22, 12.2 for control vs. FLX14, and 16.53 for FLX14 vs. FLX22).

The granule cell dematuration induced by FLX22 includes reversal of functional maturation of these cells [9]. In the matured state, the synapse between the mossy fiber, the granule cell axon, and CA3 pyramidal cells shows prominent frequency facilitation (see Figure 1A right), and this frequency facilitation is greatly reduced in the immature or dematurated granule cells [9-11]. To directly investigate the relationship between this physiological index of granule cell dematuration and the behavioral change, we made electrophysiological recordings using hippocampal slices prepared from mice whose home cage activity had been monitored. In control and FLX14-treated mice, strong frequency facilitation was induced at the mossy fiber synapse by 1 Hz stimulation (Figure 1A right and 1C right). In FLX22-treated mice that showed marked fluctuation of cage activity levels, frequency facilitation was strongly decreased (Figure 1E right), confirming the induction of the granule cell dematuration in these mice. The magnitude of mossy fiber synaptic facilitation in FLX22-treated mice was negatively correlated with CV of home cage activity levels in individual mice (Figure 1H). Thus, mice with more dematurated mossy fiber synapses exhibited larger changes in fluctuation of activity.

In some cases, we stopped administration of fluoxetine while continuously monitoring the home cage activity (Figure 2A). Although the results were variable, the activity fluctuation could be clearly observed at least up to 4 weeks after discontinuation of the fluoxetine treatment, and one mouse showed marked fluctuation only after the discontinuation (Figure 2A and 2B). Thus, the destabilization of home cage activity by FLX22 cannot be simply explained by fluctuation of acute actions or tissue concentrations of fluoxetine, but is likely caused by some plastic changes in the central nervous system. We also examined the mossy fiber synaptic transmission in mice withdrawn from FLX22, and found that frequency facilitation remained suppressed 4 weeks after the withdrawal (Figure 2C), suggesting that the granule cells were still in the dematurated state. Taken together, these results indicate that the granule cell dematuration is associated with destabilization of home cage activity of mice.

### Increased anxiety-related behaviors caused by chronic fluoxetine

**Figure 3:**
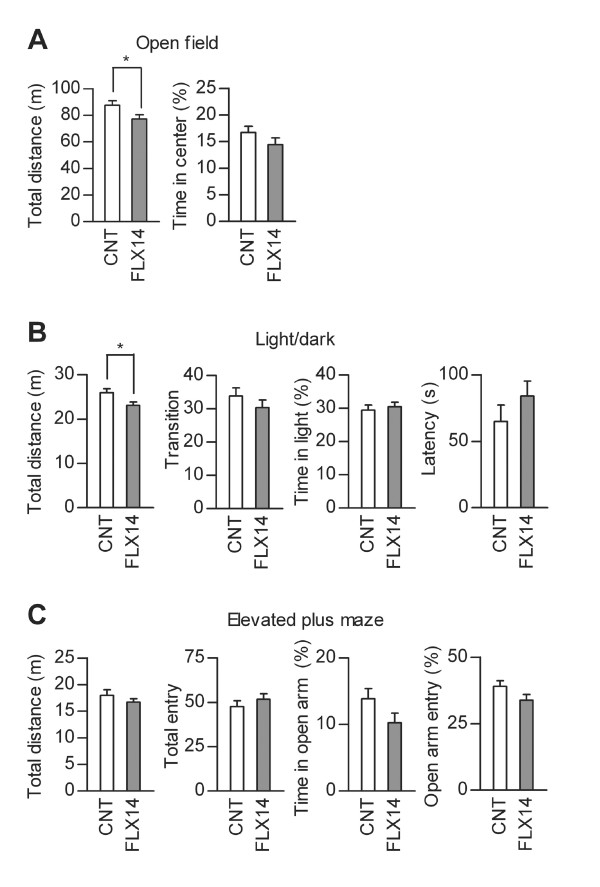
**Effects of FLX14 on locomotor activity and anxiety-related behavior**. (**A**) FLX14 reduced the total distance traveled in the open-field test (z = 2.094, *p *= 0.0363), but had no significant effect on time spent in the center of the field (z = 0.952, *p *= 0.3412, CNT: *n *= 13, FLX14: *n *= 12). Time in center is indicated as percent time spent in the center relative to the total time in the field. (**B**) FLX14 reduced the total distance traveled in the light/dark transition test (z = 2.203, *p *= 0.0276), but had no significant effects on the number of transition between two compartments (z = 0.952, *p *= 0.3408), time spent in the light box (z = 0.408, *p *= 0.6833), or latency to enter the light box (z = 1.822, *p *= 0.0685, CNT: *n *= 13, FLX14: *n *= 12). Time in light is indicated as percent time spent in the light compartment relative to the total test time. (**C**) In the elevated plus-maze test, FLX14 had no significant effects on the total distance traveled (z = 0.68, *p *= 0.4966), the total number of entries into arms (z = 0.897, *p *= 0.3693), time spent on open arms (z = 1.523, *p *= 0.1278), or entry into open arms (z = 1.768, *p *= 0.0772, CNT: *n *= 13, FLX14: *n *= 12). Time in open arms and open arm entry are indicated as percent time spent on open arms relative to the total test time and the percent number of entries into open arms relative to the total number of entries, respectively. Data are presented as mean ± s.e.m.

**Figure 4:**
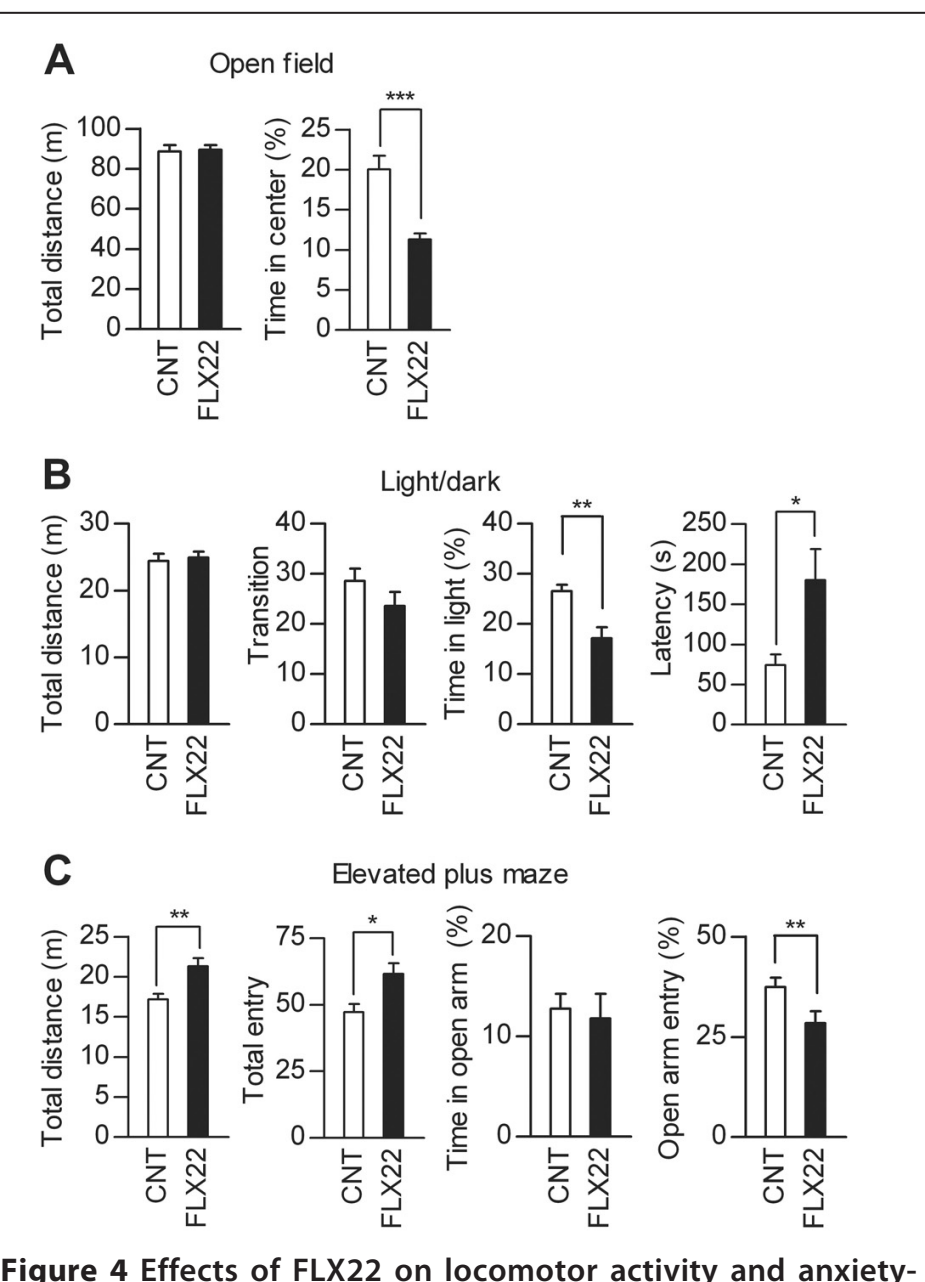
**Effects of FLX22 on locomotor activity and anxiety-related behavior**. (**A**) FLX22 reduced time spent in the center of the open field (z = 4.766, *p *< 0.0001), but had no significant effect on the total distance traveled (z = 0.08, *p *= 0.9361, CNT: *n *= 25, FLX22: *n *= 28). (**B**) FLX22 reduced time spent in the light compartment in the light/dark transition test (z = 3.185, *p *= 0.0015) and increased the latency to enter the light compartment (z = 2.217, *p *= 0.0267), but had no significant effects on the total distance traveled (z = 0.176, *p *= 0.8602) or the number of transition between two compartments (z = 0.983, *p *= 0.3253, CNT: *n *= 17, FLX22: *n *= 21). Two fluoxetine-treated mice did not enter the light compartment. (**C**) FLX22 increased the total distance traveled (z = 2.69, *p *= 0.0072) and the number of entries into arms (z = 2.465, *p *= 0.0137), and decreased relative entry into open arms (z = 2.704, *p *= 0.0069), but had no significant effect on time in open arms (z = 1.225, *p *= 0.2205) in the elevated plus-maze test (CNT: *n *= 18, FLX22: *n *= 21). Percent values are calculated as in Figure 3.

**Figure 5:**
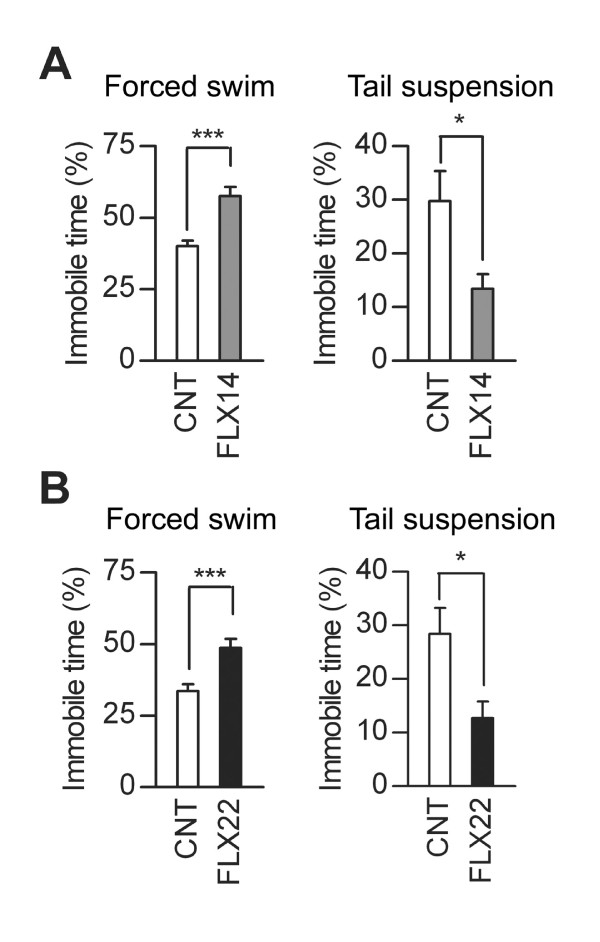
**Effects of fluoxetine on depression-related behavior**. (**A**) FLX14 increased immobile time in forced swim test (CNT: *n *= 13, FLX14: *n *= 12, z = 3.726, *p *= 0.0002) and decreased immobile time in tail suspension test (CNT: *n *= 7, FLX14: *n *= 8, U = 8, *p *= 0.0205). (**B**) FLX22 increased immobile time in forced swim test (CNT: *n *= 16, FLX22: *n *= 18, z = 3.088, *p *= 0.0009) and decreased immobile time in tail suspension test (CNT: *n *= 7, FLX22: *n *= 8, U = 8, *p *= 0.0205). Immobile time is indicated as percent time spent immobile relative to the total time monitored.

We further characterized behavioral changes caused by the fluoxetine treatments. FLX14 slightly reduced horizontal activity of mice in the open-field test (Figure 3A) and light/dark transition test (Figure 3B), but had no significant effects on indexes of anxiety-related behaviors in these tests (Figure 3A and 3B) and the elevated plus-maze test (Figure 3C). These results are similar to our previous report of behaviors of mice treated with fluoxetine at 10 mg/kg/day [12]. Effects of FLX22 on behaviors in these tests were completely different from those of FLX14. FLX22 had no suppressive effects on the locomotor activity in the open-field test (Figure 4A) and light/dark transition test (Figure 4B), induced hyperactivity in the elevated plus-maze test (Figure 4C), and consistently increased anxiety-related behaviors: FLX22 reduced time spent in the center of the open field (Figure 4A), time spent in the light box (Figure 4B), and relative entry into open arms (Figure 4C). On the other hand, FLX14 and FLX22 had similar effects on depression-related behaviors. Immobile time was increased in the forced swim test, but decreased in the tail suspension test in both conditions (Figure 5A and 5B). Taken together, these results suggest that the granule cell dematuration induced by FLX22 is associated with increased anxiety-related behaviors as well, but not with the depression-related behaviors assessed in the present study.

### Requirement of serotonin 5-HT_4 _receptor for behavioral effects of fluoxetine

**Figure 6:**
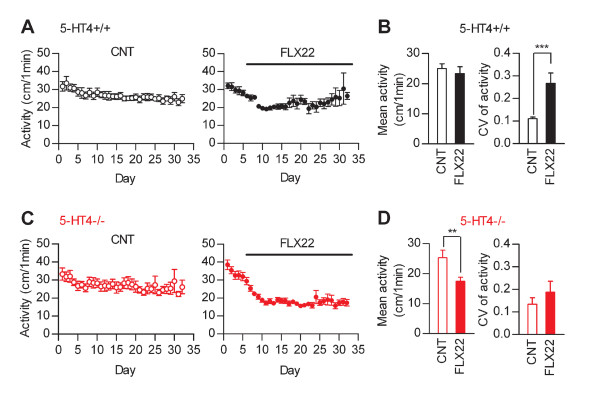
**Suppression of fluoxetine-induced behavioral destabilization in 5-HT_4_-deficient mice**. (**A**) Averaged data showing home cage activity levels of control and FLX22-treated 5-HT_4_+/+ mice (*n *= 9 each). (**B**) FLX22 increased CV of activity levels during last 2 weeks of treatments in 5-HT_4_+/+ mice (U = 5, *p *= 0.0008), but had no significant effect on mean activity levels during the same period (U = 30, *p *= 0.3865). (**C**) Averaged data showing home cage activity levels of control and FLX22-treated 5-HT_4_-/- mice (*n *= 11 each). (**D**) FLX22 reduced mean activity levels during last 2 weeks of treatments (z = 2.627, *p *= 0.0087), but had no significant effect on CV of activity in 5-HT_4_-/- mice (z = 0.591, *p *= 0.5546).

**Figure 7:**
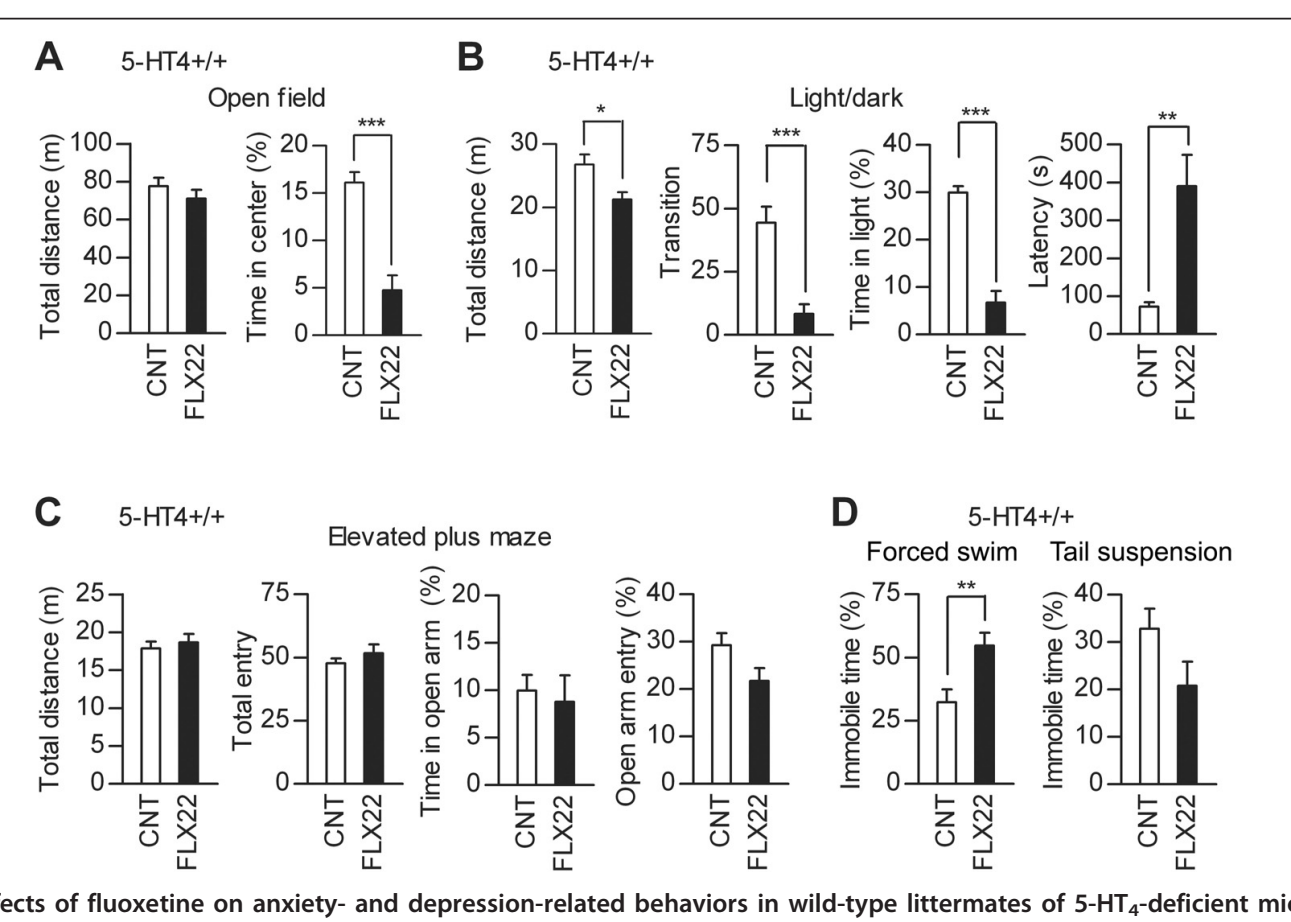
**Effects of fluoxetine on anxiety- and depression-related behaviors in wild-type littermates of 5-HT_4_-deficient mice**. (**A**) FLX22 reduced time spent in the center of the open field (U = 3, *p *= 0.0003), but had no significant effect on the total distance traveled (U = 28, *p *= 0.3154) in 5-HT_4_+/+ mice (CNT: *n *= 10, FLX22: *n *= 8). (**B**) FLX22 reduced the total distance traveled (U = 15, *p *= 0.0464), the number of transition (U = 2.5, *p *= 0.0003), and time spent in the light compartment (U = 0, *p *< 0.0001) and increased the latency to enter the light compartment (U = 6, *p *= 0.0025) in the light/dark transition test in 5-HT_4_+/+ mice (CNT: *n *= 9, FLX22: *n *= 8). Four fluoxetine-treated mice did not enter the light compartment. (**C**) FLX22 had no significant effects on the total distance traveled (U = 36, *p *= 0.7618), the total number of entries into arms (U = 31, *p *= 0.4598), time spent on open arms (z = 0.8, *p *= 0.4082), or entry into open arms (U = 20, *p *= 0.0831) in elevated plus-maze test in 5-HT_4_+/+ mice (CNT: *n *= 10, FLX22: *n *= 8). (**D**) FLX22 increased immobile time in forced swim test in 5-HT_4_+/+ mice (CNT: *n *= 10, FLX22: *n *= 8, U = 11, *p *= 0.0085), but had no significant effect in the tail suspension test (U = 19, *p *= 0.0676). Percent values are calculated as in Figure 3 and 5.

**Figure 8:**
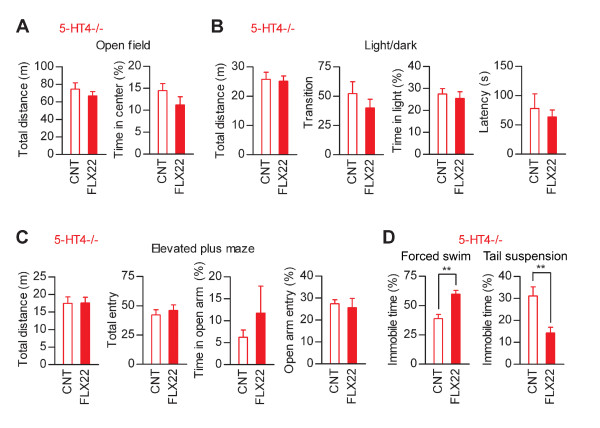
**Effects of fluoxetine on anxiety- and depression-related behaviors in 5-HT_4_-deficient mice**. (**A**) FLX22 had no significant effects on the total distance traveled (U = 23, *p *= 0.3823) or time spent in the center (U = 22, *p *= 0.3282) in the open field test in 5-HT_4_-/- mice (*n *= 8 each). (**B**) FLX22 had no significant effects on the total distance traveled (U = 30, *p *= 0.8785), the number of transition (z = 1.05, *p *= 0.2786), time spent in the light compartment (z = 0.735, *p *= 0.4418), or the latency to enter the light compartment (U = 31, *p *= 0.9591) in the light/dark transition test in 5-HT_4_-/- mice (*n *= 8 each). (**C**) FLX22 had no significant effects on the total distance traveled (U = 28, *p *= 0.7209), the total number of entries into arms (z = 0.945, *p *= 0.3282), time spent on open arms (U = 32, *p *= 1), or entry into open arms (U = 28, *p *= 0.7209) in the elevated plus-maze test in 5-HT_4_-/- mice (*n *= 8 each). (**D**) FLX22 increased immobile time in the forced swim test (*n *= 8 each, U = 4, *p *= 0.0019) and decreased immobile time in the tail suspension test in 5-HT_4_-/- mice (*n *= 8 each, U = 7, *p *= 0.007). Percent values are calculated as in Figure 3 and 5.

We have previously shown that the granule cell dematuration is attenuated in mice lacking the serotonin 5-HT_4 _receptor [9]. To further investigate the association between the granule cell dematuration and behavioral effects of fluoxetine, we carried out the same battery of behavioral tests in 5-HT_4 _receptor-deficient (5-HT_4_-/-) mice with the genetic background of C57BL/6J. As in normal C57BL/6J mice, FLX22 induced destabilization of home cage activity in the wild-type (5-HT_4_+/+) mice (Figure 6A and 6B). In 5-HT_4_-/- mice, however, FLX22 reduced mean activity levels in home cages with no significant effect on CV of activity (Figure 6C and 6D). Effects of FLX22 on other forms of behaviors in 5-HT_4_+/+ mice were also generally comparable to those in normal C57BL/6J mice: FLX22 increased anxiety-related behaviors in the open field and light/dark transition tests (Figure 7A and 7B), and increased immobile time in the forced swim test (Figure 7D). However, FLX22 did not significantly change the behaviors of 5-HT_4_+/+ mice in the elevated plus-maze test (Figure 7C) and tail suspension test (Figure 7D), and induced exaggerated anxiety-like responses in the light/dark transition test (Figure 7B). In 5-HT_4_-/- mice, FLX22 had no significant effects on behaviors in the open-field, light/dark transition and elevated plus-maze tests (Figure 8A, 8B and 8C). However, effects of FLX22 in the forced swim and tail suspension tests were similar to those in 5-HT_4_+/+ and normal C57BL/6J mice (Figure 8D), excluding the possibility that the 5-HT_4 _deficiency nonspecifically suppressed fluoxetine actions. These results further support the idea that the granule cell dematuration is associated with destabilization of home cage activity and increased anxiety-related behaviors, but not with the depression-related behaviors.

## Discussion

The present study demonstrated that chronic fluoxetine treatments induced a marked day-to-day fluctuation of activity levels of mice in familiar environments. This novel behavioral effect of fluoxetine was strongly associated with the dematuration of dentate granule cells induced by fluoxetine [9]: 1) Both effects were observed at 22 mg/kg/day, but not at 14 mg/kg/day, 2) both effects started emerging in about 2 weeks of the treatment [9], 3) both effects were suppressed in 5-HT_4 _receptor-deficient mice, and 4) the fluctuation of activity levels was negatively correlated with the magnitude of frequency facilitation at the mossy fiber-CA3 synapse, the physiological index of the state of granule cell maturation [9-11,13]. In mice heterozygous for alpha-calcium/calmodulin-dependent protein kinase II (alpha-CaMKII+/- mice), the dentate granule cells stay in the immature state even in adults, and these mice also show strongly reduced frequency facilitation at the mossy fiber synapse and aberrant home cage behaviors with large periodic changes in activity levels [11]. These lines of evidence raise the possibility that the abnormality in the state of granule cell maturation causes instability in activity of mice in familiar environments. Since lesion of the hippocampus consistently causes hyperactivity in home cages [14-16], the hippocampus certainly plays an essential role in regulating activity levels in familiar environments. The dematurated granule cells in the fluoxetine-treated mice and immature granule cells in alpha-CaMKII+/- mice exhibit similar and characteristic changes in their functional properties [9,11]. Higher excitability in these granule cells would allow propagation of more cortical excitation into the hippocampus, and the reduced mossy fiber synaptic facilitation would impair the instructive role of the mossy fiber input in the induction of associative synaptic plasticity in the CA3 region [17,18]. In addition, activity-dependent expression of the immediate early gene c-fos is nearly completely suppressed in both dematurated and immature granule cells in these mice [9,11]. These diverse changes in granule cell functioning would substantially modify activity of the hippocampal neuronal circuit [13], thereby quite likely affecting hippocampus-dependent behaviors such as locomotor activity in home cages. It should be noted that immaturity or dematuration of the dentate granule cells can be assessed not only by the electrophysiological method, but also by a real-time PCR or histochemical analysis of expression of molecular markers such as calbindin and tryptophan 2,3-dioxygenase [9,11,19-21]. These molecular markers would be useful in examining a possible association between abnormality in the state of granule cell maturation and behavioral phenotypes in other mutant or drug-treated mice. The aberrant behavioral effect of fluoxetine observed here might be related to adverse effects of fluoxetine and other SSRIs in humans. SSRIs can induce switching to mania or mood destabilization, especially in patients with bipolar disorder [1,5], and the switching to mania can be observed even after discontinuation of SSRI medication [4]. Since the destabilization of mouse behaviors were observed at the dose higher than the clinical therapeutic dose of fluoxetine and suggested optimal dose for mice [22], this effect may not be directly relevant to clinical symptoms or behavioral changes in humans. Although further studies are necessary to clarify the clinical relevance of the present result, our finding indicates that the SSRI fluoxetine has the potential to induce rapid changes in the behavioral state during chronic treatments and after discontinuation of the treatments.

The granule cell dematuration was associated with the increased anxiety-related behaviors in addition to the behavioral destabilization. Some previous studies have also shown that chronic fluoxetine can increase anxiety-related behaviors [23,24]. In our studies using adult mice, the anxiogenic-like effect of fluoxetine was observed at 22 mg/kg/day, but not at 14 mg/kg/day or 10 mg/kg/day [12]. On the other hand, Oh et al. (2009) have shown the anxiogenic-like effect of fluoxetine at as low as 3 mg/kg/day in juvenile mice [24]. In their study, fluoxetine was administered from the age of 2 through 6 weeks. The magnitude of frequency facilitation at the mossy fiber synapse reaches the mature level at the age of 3 or 4 weeks in mice [9,10]. Therefore, at the beginning of such treatment period, many granule cells are supposed to be in the immature state. It is possible that the difference in the state of maturation of granule cells contributes to the higher potency of fluoxetine in increasing anxiety-related behaviors in young mice. While the present study shows the association between the granule cell dematuration and increased anxiety-related behaviors, alpha-CaMKII+/- mice, which have immature granule cells, exhibit greatly reduced anxiety-related behaviors [11]. This apparent discrepancy could be due to differences in neuronal functions in brain regions other than the dentate gyrus. Alternatively, it might be ascribed to differences in physiological properties between the fluoxetine-treated dematurated granule cells and the immature granule cells in alpha-CaMKII+/- mice. The immature granule cells in alpha-CaMKII+/- mice are more easily excited by depolarizing current injection than the fluoxetine-treated dematurated granule cells, but show a faster decline of firing during prolonged depolarization [9,11]. Therefore, these two types of granule cells could behave in a quite different way in some occasions, thereby differentially contributing to the same hippocampus-dependent behavior.

Our results demonstrated the involvement of the 5-HT_4 _receptor in some of behavioral effects of fluoxetine. This finding is in line with the previous report showing that subchronic administration of 5-HT_4 _receptor partial agonists had antidepressant-like behavioral effects in rats [25]. The 5-HT_4 _receptor is abundantly expressed in the dentate granule cells [26], mediates serotonin-induced synaptic potentiation at the mossy fiber synapse [9,12], and is essential for the granule cell dematuration induced by FLX22 [9]. In 5-HT_4_-/- mice, FLX22 had no significant effects on fluctuation of home cage activity levels or anxiety-related behaviors. Since we did not observe any obvious difference in behaviors between the 5-HT_4_+/+ and 5-HT_4_-/- mice of the fluoxetine-free control groups, it is unlikely that behavioral effects of fluoxetine were masked by preexisting behavioral abnormalities in the mutant mice. In addition, FLX22 was still effective in changing some forms of behaviors in 5-HT_4_-/- mice: FLX22 changed the depression-related behaviors as in 5-HT_4_+/+ and normal C57BL/6J mice and significantly reduced activity levels in the home cages instead of inducing destabilization of activity. Thus, the 5-HT_4 _deficiency does not have nonselective suppressing effects on fluoxetine actions, supporting the specific association between fluoxetine-induced behavioral and cellular changes that are attenuated in the mutant mice. Compan et al. (2004) have reported a significant decrease in open-field locomotor activity in 5-HT_4_-/- mice [27]. This discrepancy may be due to differences in experimental conditions, most probably the difference in the genetic background of the mutant mice: 129/Sv in Compan et al. (2004) vs. C57BL/6J in our study.

## Conclusions

Chronic fluoxetine treatments can induce destabilization of home cage behaviors in normal mice that is characterized by occasional switching between hyperactivity and hypoactivity within a few days. This behavioral destabilization was attenuated in mice lacking the serotonin 5-HT_4 _receptor and tightly associated with the fluoxetine-induced dematuration of the dentate granule cells. Based on these results, we propose that the dematuration of dentate granule cells can be a candidate cellular process underlying switching-like changes in the behavioral state that are associated with SSRI treatments.

## Methods

### Drug Treatment

Male C57BL/6J mice were singly housed from the age of 8 weeks in the institutional standard condition (14:10 light/dark cycle; lights on at 6:00 A.M. through 8:00 P.M.) at 23 ± 1°C with food and water available ad libitum. Following 1 week of acclimation, fluoxetine hydrochloride (Wako Pure Chemical Industries, Ltd., Osaka, Japan) was orally applied at a dose of 14 or 22 mg/kg/day. In order to minimize stress associated with drug administration, fluoxetine was dissolved in the drinking water. The fluoxetine solutions were prepared everyday, and concentrations of fluoxetine were determined for individual mice based on the water consumption during preceding 24 h and the body weight measured every other day [9]. Saccharin (0.2%) was included in the fluoxetine solution to keep water intake comparable to the baseline [9]. Control mice were given water with or without saccharin, and all data were pooled. After 4 weeks of fluoxetine treatments, behavioral tests were started, and fluoxetine treatments were continued during the tests. The 5-HT_4 _receptor heterozygous mutant mice (strain name: B6.129P2-Htr4 < tm1Dgen >/J) were purchased from the Jackson Laboratory (Bar Harbor, ME, USA). These mice had been backcrossed to the C57BL/6J background for 10 generations. Male wild-type (5-HT_4_+/+) and homozygous mutant (5-HT_4_-/-) littermates from heterozygous mating were treated with fluoxetine and used for experiments. There was no significant difference in the actual doses of fluoxetine administered to mice between the genotypes. All procedures were approved by the institutional Animal Care and Use Committee.

### Home cage activity monitoring

Mice were singly housed in the cage (15 × 25 × 30 cm) equipped with an infrared video camera at the top, and locomotor (horizontal) activity in the cage was continuously monitored. Outputs from the cameras were fed into a personal computer. Images were captured at a rate of one frame per second, and the distance traveled per minute was analyzed online using software based on the public domain ImageJ (ImageJ HC8; O'Hara and Co., Ltd., Tokyo, Japan). Since activity of mice during the light period could be affected by external stimuli such as noise made by workers in the animal facility, the activity during the dark period (nocturnal activity) was evaluated in quantitative analyses.

### Behavioral experiments

Mice were transferred to a behavioral testing room and allowed to acclimatize to the environment of the room for at least 1 h 30 min before starting behavioral tests. All tests were performed between 13:30 P.M. and 18:00 P.M. The tests were sequentially performed on different days in the order of the open-field test, light-dark transition test, elevated plus-maze test, forced swim test and tail suspension test. Only one test was conducted for each mouse in a day. Room temperature was kept at 24 ± 0.5 °C. To minimize olfactory cues from the previous trial, each apparatus was wiped and cleaned with a hypochlorous acid solution (~15 ppm, pH 5 - 6.5) before each test except the forced swim test and the tail suspension test.

The open-field test was carried out to examine locomotor activity and anxiety-related behavior in a novel environment. The open-field apparatus composed of opaque white walls and a floor (50 × 50 × 50 cm) was illuminated at an intensity of 40 lux. Each mouse was placed in the center of the open-field arena, and then locomotor (horizontal) activity was monitored for 20 min via a CCD camera positioned above the apparatus. The ambulatory distance and relative time spent in the central zone were measured. To calculate relative time spent in the center, the floor of the apparatus was divided into 25 squares and time spent in the central nine squares was measured. Fluoxetine did not significantly affect vertical activity assessed by measuring the number of infrared photobeam interruption. All records were stored on a PC and analyzed using software based on the public domain ImageJ (ImageJ OF; O'Hara and Co., Ltd., Tokyo, Japan).

The light/dark transition test was carried out to assess anxiety-related behavior. The apparatus for this test was composed of a box (21 × 42 × 25 cm) divided into two compartments of equal size. One compartment was brightly illuminated at an intensity of 600 lux and a CCD camera was equipped on the ceiling, whereas the other was not illuminated (8 lux) and an infrared camera was equipped on the ceiling. The partition dividing the compartments had an opening (5 × 3 cm) with a sliding door through which mice could move from one compartment to the other. Single mice were put into the dark compartment and the sliding door was automatically opened after 5 s. The behavior of mice was monitored for 10 min. The total number of transitions between chambers, time spent in each side, the first latency to enter the light side and the distance traveled in each side were recorded automatically and analyzed using software based on the public domain ImageJ (ImageJ LD2, O'Hara & Co., Ltd., Tokyo, Japan). When mice did not enter the light compartment, the latency was considered 600 s.

The elevated plus-maze test was carried out to examine anxiety-related behavior. The apparatus consisted of a central platform (5 × 5 cm), two opposed open arms (25 × 5 cm) and two opposed closed arms of the same size, but with 15-cm-high opaque walls. The edges of the open arms were raised 0.25 cm to avoid falls of mice. The floor of each arm was made of white plastic. The apparatus was elevated to a height of 50 cm above the floor and illuminated at an intensity of 40 lux. At the beginning of each test, one of the open arms was blocked by a transparent obstacle and each mouse was placed on the central platform, facing the blocked open arm. After the mouse entered either of the closed arms, the block was removed and the test was started. Activity of mice was monitored for 10 min via a CCD camera positioned above the apparatus. The time spent in each arm, the number of entries into each arm and the ambulatory distance were recorded and analyzed using software based on the public domain ImageJ (ImageJ EP, O'Hara & Co., Ltd., Tokyo, Japan).

The forced swim test was carried out to assess depression-related behavior. The apparatus consisted of a transparent plastic cylinder (22 cm in height and 12 cm in diameter) placed in a box (30.5 × 40 × 40 cm). The cylinder was filled with water (25°C) up to a height of 10 cm and was illuminated from the bottom of the box. Each mouse was placed into the cylinder and activity was monitored for 15 min via a CCD camera mounted on the top of the box. Immobile time except for first 2 min was measured. The cylinder was refilled with clean water after each test. All records were stored on a PC and analyzed using software based on the public domain ImageJ (ImageJ PS4, O'Hara & Co., Ltd., Tokyo, Japan).

The tail suspension test was carried out to examine depression-related behavior. The tip (1 cm) of mouse tail was securely fastened with adhesive tape to a metallic plate. The plate was hung from the ceiling of a test box (30.5 × 40 × 40 cm). Immobile time was measured for 6 min with a CCD camera mounted on the side of the box. All records were stored on a PC and analyzed using software based on the public domain ImageJ (ImageJ PS4, O'Hara & Co., Ltd., Tokyo, Japan).

### Electrophysiology

Mice were decapitated under deep halothane anesthesia and both hippocampi were isolated. Transverse hippocampal slices (380 μm) were cut using a tissue slicer in ice-cold sucrose-containing saline composed of (in mM): sucrose 72, NaCl 80, KCl 2.5, NaH_2_PO_4 _1.0, NaHCO_3 _26.2, glucose 20, CaCl_2 _0.5, MgCl_2 _7 (equilibrated with 95% O_2 _/5% CO_2_). Slices were then incubated for 30 min at 30°C and maintained in a humidified interface holding chamber at room temperature (24 - 27°C) before recordings. Electrophysiological recordings were made in a submersion-type chamber maintained at 27.0 - 27.5°C and superfused at 2 ml/min with saline composed of (in mM): NaCl 125, KCl 2.5, NaH_2_PO_4 _1.0, NaHCO_3 _26.2, glucose 11, CaCl_2 _2.5, MgCl_2 _1.3 (equilibrated with 95% O_2 _/5% CO_2_). Field excitatory postsynaptic potentials (fEPSPs) arising from the mossy fiber synapses were evoked by electrical stimulation delivered to the dentate granule cell layer and recorded from the stratum lucidum of CA3 using a glass pipette filled with 2 M NaCl. The amplitude of fEPSPs was measured on analysis as described [28]. A criterion used to identify the mossy fiber input was more than 85% block of fEPSP amplitude by an agonist of group II metabotropic glutamate receptors, (2 S,2'R,3'R)-2-(2',3'-dicarboxycyclopropyl)glycine (1 μM) (Tocris Bioscience, Bristol, UK). Single electrical stimulation was delivered at a frequency of 0.05 Hz for baseline recordings and frequency facilitation was examined at 1 Hz. All recordings were made using a Multiclamp 700B amplifier (Molecular Devices, Sunnyvale, CA, USA), filtered at 2 kHz and stored in a personal computer via an interface (digitized at 5 - 10 kHz).

### Statistics

The number of data (*n*) represents the number of mice. Since some data from drug-treated mice did not distribute normally, nonparametric tests were used to evaluate statistical significance. The two-tailed Mann-Whitney test and Dunn's multiple comparison test were used to compare two groups and three groups or more, respectively, with the significance level *P *< 0.05. All statistical tests were performed using GraphPad Prism version 3.03 for Windows (GraphPad Software, San Diego, CA, USA).

## Competing interests

The authors declare that they have no competing interests.

## Authors' contributions

KK designed the study, carried out electrophysiological experiments, analyzed the data, and drafted the manuscript. YI carried out behavioral experiments, analyzed the data, and helped to draft the manuscript. HS participated in the design of the study and helped to draft the manuscript. All authors read and approved the final manuscript.
